# Nurses' experiences of an outreach interprofessional mental health service for nursing homes: a qualitative descriptive study

**DOI:** 10.1111/jpm.12847

**Published:** 2022-07-18

**Authors:** Karin Fuchs, Samuel Vögeli, Dominik Schori, Daniela Händler‐Schuster

**Affiliations:** ^1^ Institute of Nursing, School of Health Sciences Zurich University of Applied Sciences Winterthur Switzerland; ^2^ Directorate of Nursing, Therapies and Social Work University Hospital of Psychiatry Zurich Zurich Switzerland; ^3^ Department Nursing Science and Gerontology Private University of Health Sciences, Medical Informatics and Technology (UMIT TIROL) Hall in Tyrol Austria; ^4^ School of Nursing, Midwifery and Health Practice, Faculty of Health Te Herenga Waka ‐ Victoria University of Wellington Wellington New Zealand

**Keywords:** geriatric psychiatry, health services research, interprofessional relations, mental disorders, mental health services, nursing homes, psychiatric nursing

## Abstract

**What is known on the subject?:**

- Treatment and mental health care in familiar environments are beneficial for older people experiencing mental health issues. But there are not enough qualified and specialized nurses who can meet the complex needs of nursing home residents experiencing mental health issues.
- The University Hospital of Psychiatry Zurich, Switzerland, established an outreach interprofessional mental health service to foster the care for residents experiencing mental health issues in nursing homes. Based on existing studies, little can be said about whether nurses in nursing homes find these types of services helpful.

**What does this paper add to existing knowledge?:**

- Nurses in nursing homes caring for residents experiencing mental health issues felt relieved by having inclusive support from the interprofessional mental health service. Nurses appreciated the mental health team and felt accompanied and more confident in their daily work.
- Results showed that nurses wanted to be included in the care and treatment processes and to work as partners on an equal footing with the mental health team.

**What are the implications for practice?:**

- Outreach interprofessional mental health services for nursing homes should take into account nurses' views and professional experience, and value and respect their role as nurses.
- Outreach interprofessional mental health services for nursing homes should offer further training in psychiatric nursing, include an accessible contact person in the team, and develop clear processes and responsibilities.

**Abstract:**

**Introduction:**

Outreach interprofessional mental health services for nursing homes can increase the quality of care for residents experiencing mental health issues but research on how nurses in nursing homes experience such a service is lacking worldwide.

**Aim:**

To describe how nurses experience the involvement of an outreach interprofessional mental health team in the care for older people experiencing mental health issues in nursing homes and to identify barriers to and facilitators of interprofessional collaboration.

**Method:**

Qualitative descriptive analysis based on 13 semi‐structured interviews. Framework analysis and complex adaptive systems theory were applied.

**Results:**

One core theme with two main categories: Nurses *experienced relief from burden through inclusive support* provided by the mental health team. Main categories were *feeling accompanied and confident as a nurse* and *partnership‐based collaboration*.

**Discussion:**

Results showed for the first time that nurses felt supported by the mental health team and were encouraged to find new ways of coping with challenging situations.

**Implications for Practice:**

To empower nurses, mental health teams should take into account nurses' perceptions in the treatment process, value and respect their role as nurses, transfer knowledge in both formal and informal settings, establish a steady and reliable contact person, and define processes and responsibilities.

## 1 INTRODUCTION

Mental health care for nursing home residents faces several challenges. Three of these challenges are particularly relevant for the present study: First, residents experiencing mental health issues often have behavioural issues, such as agitation or aggressiveness, which can lead to an increased workload for nursing staff (Collet et al., 2018; van den Brink et al., 2017; Vogel et al., 2017). Evidence suggests that work stress is positively associated with intention to leave (Gaudenz et al., 2017; Kuo et al., 2014; Stewart et al., 2011). Second, nursing staff in Swiss nursing homes are usually less qualified compared to hospital and home care staff, making it difficult to provide specialized nursing care for residents experiencing mental health issues. The skill mix in Swiss nursing homes consists of 25% nurses, 35% healthcare assistants and 40% nursing aides (Andreani, 2020). Third, many nurses leave their profession early in their life course (Marquis & Andreani, 2020; Merçay et al., 2016). As a result, nursing homes face difficulties in recruiting qualified and experienced nursing staff.

Nursing staff shortages together with complex healthcare needs of residents experiencing mental health issues likely put the quality of care at risk (McGarry et al., 2019). Previous evidence suggests that integrating mental health professionals and working in interprofessional teams can increase the quality of care for residents experiencing mental health issues and improve outcomes of care (Kilpatrick et al., 2020; Klug et al., 2019; van der Wolf et al., 2019). Also, research suggests that specialized interprofessional mental health services reaching out to nursing homes fill a gap in care for older people experiencing mental health issues (Fischer et al., 2011; Koekkoek et al., 2016; Stocker et al., 2018).

**FIGURE 1:**
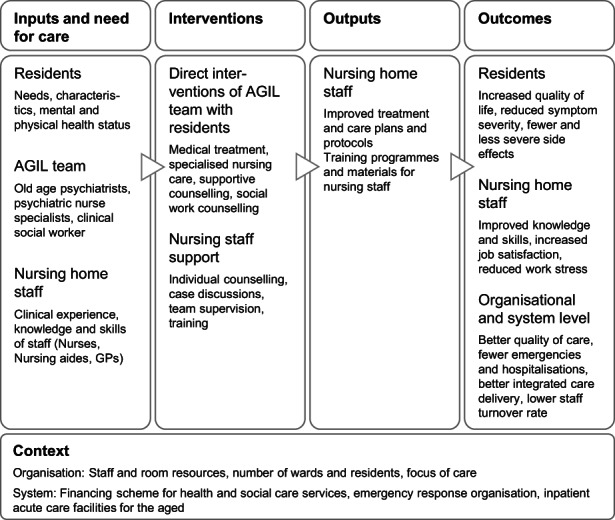
Key components and intended outcomes of AGIL

In 2013, the Clinic for Old Age Psychiatry of the University Hospital of Psychiatry Zurich, Switzerland, established an outreach interprofessional mental health service (we use the acronym AGIL that refers to the German name of the service). The target population was people aged 65 and older experiencing mental health issues and their relatives living in their own home or in nursing homes in the metropolitan area of Zurich. Old age psychiatrists, psychiatric nurse specialists and a clinical social worker were part of the AGIL team. Services included medical treatment, case discussions, specialized nursing care and social work counselling, as well as team supervision, training and counselling of staff in nursing homes. Figure 1 shows a logic model of the key components and intended outcomes of AGIL (MacDonald, 2018). Services such as AGIL are not implemented widely in the Canton of Zurich or in other parts of Switzerland.

Research evaluating outreach interprofessional mental health services is scarce. Two previous studies showed that interprofessional mental health services for nursing homes may foster the treatment of residents experiencing mental health issues and support nursing staff (Fischer et al., 2011; Koekkoek et al., 2016). To our knowledge, there is no research, national or international, on how nurses in nursing homes experience the involvement of such services in the care for older people experiencing mental health issues. Also, little is known about the barriers and facilitators that are present in the collaboration between nursing staff and interprofessional teams of outreach mental health services. Such findings are needed to further develop AGIL and other comparable outreach interprofessional mental health services that aim to improve the care for residents in nursing homes experiencing mental health issues.

We aimed to describe how nurses experience the involvement of the AGIL team in the care for older people experiencing mental health issues in nursing homes and to identify barriers and facilitators in the collaboration between nursing staff and the AGIL team.

## 2 METHODS

### 2.1 Study design

We applied a qualitative descriptive design based on framework analysis (Ritchie & Spencer, 1994; Goldsmith, 2021) to explore nurses' experiences with the involvement of the AGIL team. Framework analysis is a pragmatic approach that originated in applied political science and has been increasingly used in health research in recent years (Goldsmith, 2021). We chose complex adaptive systems (CAS) theory as the theoretical framework. CAS are characterized by relationships between different actors that change continuously through non‐linear interactions (McDaniel et al., 2009). Using CAS theory helped the first author to focus on interactions and context during data collection and analysis, and to reflect on tensions arising during the research process (McDaniel et al., 2009).

We based our research on four quality criteria: utility, feasibility, accuracy and propriety (German Society for Evaluation [DeGEval], 2019; Kidder & Chapel, 2018). The first author collected, analysed and interpreted the data as part of her master's thesis in nursing science. Regular supervision from the last author and exchanges with members of her research group supported the quality and credibility of the research process.

### 2.2 Context and setting

In Switzerland, long‐term care for elderly people is regulated on the cantonal level. Zurich is in the German‐speaking part of Switzerland, where nursing homes are decentralized and run by municipal and private institutions (Rodrigues & Nies, 2013). Nursing homes are diverse in size, location and focus of care. Basic medical care is provided by general practitioners (GPs) in private practice who give consultations in nursing homes, including psychiatric medical treatment. The costs of medical consultations and nursing care are covered by federally mandated universal health insurance (Schneeberger & Schwartz, 2018). The costs of social care are paid by the residents and, if residents do not have sufficient means to cover these costs, by the municipality (Rodrigues & Nies, 2013).

### 2.3 Recruitment and participants

At the time of recruitment, AGIL collaborated with 43 nursing homes. The size of the nursing homes ranged from residential groups with around 10 places to larger facilities with around 110 places. The nursing homes delivered generalized or specialized long‐term care. We included managers in the sample due to their significant influence on the design and quality of care (Asante et al., 2021; Backman et al., 2018). To include perspectives from various settings, we applied a purposive sampling strategy and recruited 13 participants from 12 nursing homes. Inclusion criteria for participants were direct cooperation with AGIL in the last 12 months, employment in the respective nursing home for at least six months, and having at least one year of professional experience in long‐term care. Table 1 shows the characteristics of the participants.

**TABLE 1:** Characteristics of participants (*n* = 13)

**Age** [years], mean (SD)	41.5 (9.6)
**Gender**	
Female	9
Male	4
**Education level**	
Healthcare assistant^a^	2
Registered nurse	10
Academic education in nursing	1
**Work experience in nursing** [years], mean (SD)	
Total work experience	20.0 (9.3)
Long‐term care (*n* = 13)	16.2 (9.7)
Psychiatric nursing (*n* = 7)	2.2 (3.8)
**Workload**	
Full‐time (36–42 h/week)	11
Part‐time (24–34 h/week)	2
**Length of current employment**[years], mean (SD)	3.9 (2.7)
**Position**	
Nurse manager	8
Nurse without management duties	5
**Collaboration with professional group of the AGIL team**	
Old age psychiatrists and psychiatric nurse specialists	10
Old age psychiatrists only	2
Psychiatric nurse specialists only	1
Social workers	0
**Kinds of services used**	
Participation in case discussions	9
No participation in case discussions	4
Participation in training	3
No participation in training	10

^a^ Federal Diploma of Vocational Education and Training (upper secondary education).

### 2.4 Ethics

This study does not fall under the remit of the Swiss Human Research Act because no health‐related personal data were obtained or processed. The responsible ethics committee issued a declaration of non‐competence. Participants received verbal and written information about the study and gave written informed consent. Withdrawal was possible at any time. The study presented no additional risks to the participants or the residents of the nursing homes.

### 2.5 Data collection

Data were collected with semi‐structured individual interviews, field notes and a questionnaire to obtain sociodemographic data. We developed the interview guide based on the literature and used the principles of collecting, checking, sorting and subsuming to generate the interview questions (Helfferich, 2011). Table 2 lists the interview prompts. Interviews were conducted between September and December 2020 and lasted between 28 and 66 minutes. The average duration was 40 minutes. Eleven interviews were conducted face‐to‐face in the nursing homes. Due to rising COVID‐19 cases during the period of data collection (Riou et al., 2021), two interviews were conducted by phone.

**TABLE 2:** Interview prompts

What has been your experience working with AGIL?
From your point of view, what has changed through working with AGIL?
In what ways would you like to have support in the care of residents experiencing mental health issues?
What do you find challenging about caring for residents experiencing mental health issues?

### 2.6 Data analysis

The interviews were recorded and transcribed verbatim (Dresing & Pehl, 2011). To protect personal data, the names of the participants and the nursing homes where they were employed were coded in the transcripts (DeGEval, 2019). Transcripts were checked against the audio recordings. Data analysis involved five steps: *familiarization*, *identifying a thematic framework*, *indexing*, *charting* and *mapping and interpretation* (Ritchie & Spencer, 1994). *Familiarization* included listening to the interviews, transcribing them and reading them repeatedly. *Identifying a thematic framework* included open coding of three transcripts and developing a first thematic framework. *Indexing* included coding all transcripts and revising the thematic framework. *Charting* included writing a summary of each subtheme for each participant and adding quotations to the summaries. *Mapping and interpretation* included combining all charts to identify concepts across themes (Ritchie & Spencer, 1994). Memos were written to record thoughts and connections in the research process. We consulted memos and field notes to ensure that the main message of the interviews was reflected in the final categories (Ritchie & Spencer, 1994), and we additionally considered sociodemographic data to ensure the accuracy of the results (DeGEval, 2019). We used MAXQDA 2020 to analyse the data (VERBI Software, 2019).

## 3 FINDINGS

**FIGURE 2:**
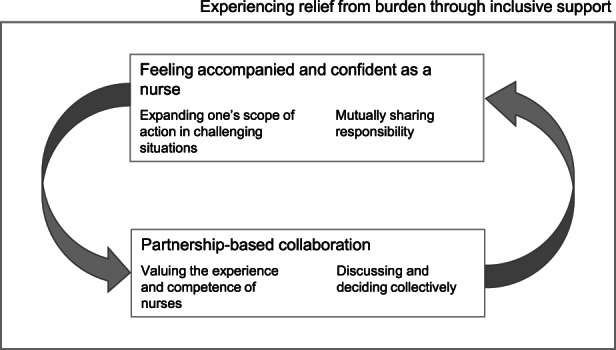
Main categories and subthemes of the analysis

The analysis revealed one core theme that contains two main categories. The core theme shows that nurses *experienced relief from burden through inclusive support* when the AGIL team was involved in the care for older people experiencing mental health issues. The two main categories relating to the core theme were *feeling accompanied and confident as a nurse* and *partnership‐based collaboration*. We found that the categories were interdependent of each other and that each category included two subthemes. Figure 2 shows the main findings.

Overall, the participants experienced relief from burden when they were involved in the treatment process. They wanted to share responsibility mutually. The participants were committed to find ways of coping with challenging situations themselves. It was important to them to be valued and respected as nurses by members of the AGIL team.

### 3.1 Feeling accompanied and confident as a nurse

The first main category included two subthemes: *expanding one's scope of action in challenging situations* and *mutually sharing responsibility* with the AGIL team.

#### 3.1.1 Expanding one's scope of action in challenging situations

This subtheme shows that participants were committed to expanding their scope of action in challenging situations. They did so by extending their knowledge and integrating the perspective of the AGIL team.

##### Challenging situations

All participants encountered challenging situations when caring for residents experiencing mental health issues. Most frequently mentioned were situations in which residents did not behave as the participants expected, and situations in which the participants felt that residents were unwilling or unable to engage in nursing interventions. About half of the participants reported having experienced verbal or physical aggression by residents. Further phenomena that were reported included suspected but unknown traumatic experiences in the biography of the residents, as well as suicidality and agitation due to dementia. Some participants mentioned caring for residents with alcohol dependence, depression or personality disorders. The participants reported that their nursing staff lacked knowledge and experience in psychiatric nursing. They also reported that staff had difficulties reaching residents emotionally, especially when the residents were cognitively impaired or when information about the biography or condition of the residents was lacking. According to the participants, residents' health issues have become more complex in recent years. These issues possibly contribute to the considerable number of challenging situations that participants have been facing:

Because at some point you reach your limits, where you just do not know anymore, what do I do right? What measures do I have to take?(Participant 8)

##### Extending knowledge

Participants appreciated that the AGIL team passed on its knowledge about how to find new ways to cope with challenging situations during medical visits, training units, or case discussions. In addition to the formal encounters, participants appreciated the possibility to ask questions in informal settings. In the participants' view, this professional exchange fostered partnership‐based collaboration (see below, main category *partnership‐based collaboration*). The participants were keen to learn more about psychiatric nursing, especially about how to handle difficult situations to expand their scope of action and manage critical situations on their own. This demand for knowledge translation corresponds with the results shown in Table 1: seven out of the 13 participants declared that they had previous experience in psychiatric nursing (mean duration: 2.3 years). Some of the participants felt that they did not receive enough training from the AGIL team and expressed a need for additional training units.

##### Integrating an outside perspective

Integrating the perspective of the AGIL team helped several participants to get a broader view of a situation and enabled them to approach situations in a more reflective way. The participants became aware that they had done little, if anything, wrong, which seemed to be important to them. Also, they acquired new strategies to deal with challenging situations, as shown in the following quote:

It was good that he [psychiatrist nurse specialist of the AGIL team] came here and did training with us, where we could ask our questions and feel more secure that we are doing something right, but that we could also make improvements.(Participant 1)

#### 3.1.2 Mutually sharing responsibility

This subtheme shows that participants felt relieved to mutually share responsibility for the care for the residents. The AGIL team facilitated sharing responsibility by being accessible, working continuously with the participants and easing the workload of the participants.

##### Accessibility

All participants mentioned that they felt accompanied by the AGIL team, mainly when team members were easily accessible. When the AGIL team was not readily accessible, the participants were concerned that this would impede them in providing appropriate care. About half of the participants mentioned that they generally had trouble during psychiatric emergencies in getting support from an emergency psychiatrist within a reasonable time and had to handle emergency situations on their own. Some participants tried to reach out to the AGIL team in such cases. This suggests that they were probably not aware that responding to emergencies is not part of AGIL's remit. However, when participants were able to reach the AGIL team in critical situations, they were guided in providing the necessary care and treatment, which helped to prevent emergencies:

Because in the past, the biggest stress factor was that we had situations that were extremely difficult and stressful for us, for the resident himself who was affected as well as for the rest of the residents. And what is extremely difficult is that you try to reach a doctor, and no one is available anywhere. You just have the feeling that you are alone in the situation, you cannot get any further, and the situation is no longer bearable. And that is quite bad. And that has never really been the case with me in this project.(Participant 2)

##### Continuity of staff

The participants reported that having access to a reliable AGIL contact person made them more comfortable to share responsibility with him or her. Two of the participants considered it a barrier that the contact person changed from time to time or, in one case, was not known at all. Changes of the contact person occurred because some of the nursing homes were attended by assistant physicians who rotate yearly. Moreover, senior physicians of the AGIL team knew the distinctive characteristics of the nursing homes and were able to establish trusting relationships with the participants, which facilitated developing a partnership‐based collaboration (see below, main category *partnership‐based collaboration*).

##### Easing the workload

Participants reported to have limited time to engage in conversations with residents. Nearly all participants related this to staff shortages in long‐term care. The psychiatric nurse specialist of the AGIL team supported the participants by engaging in daily activities or conversations with residents. Participants in management positions felt relieved because the AGIL team organized and led case discussions. One participant described experiencing the support as follows:

We experienced it as very liberating during this time. It was good for the team that we could just let go and say: Look, she will get another visit tomorrow; someone will take care of her again tomorrow. We could, how shall I say, not let go, but we could put it in other hands. And I think that did the team some good as well.(Participant 12)

Half of the participants wished that the AGIL team would visit more often. More frequent visits might strengthen continuity and promote positive aspects of the involvement of AGIL, such as a reduced workload and emotional relief.

### 3.2 Partnership‐based collaboration

The second main category shows that participants wanted to work as partners with the AGIL team. It included two subthemes: *valuing the experience and competence of nurses* and *discussing and deciding collectively* as an interprofessional team.

#### 3.2.1 Valuing the experience and competence of nurses

This subtheme shows that it was important to the participants to be valued as nurses. This finding was reflected in statements of the participants as they related their own strategies and work experience in caring for residents experiencing mental health issues. This subtheme includes receiving positive feedback about their work and coping skills.

##### Participants' own strategies and work experience

Participants emphasized their own strategies and work experience in caring for residents experiencing mental health issues. Strategies included conducting self‐organized case discussions or trainings, supporting each other professionally and emotionally within a team, implementing nursing interventions, and intensifying care. It seemed important to the participants that their effort was acknowledged by the AGIL team. Two participants expressed this as follows:

We do not always need someone from outside.(Participant 3)

We also know a little bit about how to handle things.(Participant 6)

The participants shared their experiences regarding the care for residents experiencing mental health issues at the institutional and societal levels. Most participants observed that residents experiencing mental health issues do not always receive adequate care. Some participants emphasized that society and healthcare institutions should better address the care for residents experiencing mental health issues:

I think it [AGIL] should be offered nationwide, because the need is there; the need is high. I know that; I have much experience. It is very supportive to have such services and to help the whole system not to fall off the rails.(Participant 11)

##### Receiving positive feedback

The participants appreciated receiving positive feedback about their work and coping skills from the AGIL team. The positive feedback contributed to a feeling of being relieved in challenging situations. Also, feeling valued and respected as nurses seemed to be fundamental for the participants to accept the support of the AGIL team and to be able to expand their scope of action (see above, subtheme *expanding one's scope of action*).

#### 3.2.2 Discussing and deciding collectively

This subtheme shows that participants wanted to discuss and decide issues collectively, as members of an interprofessional team. In the participants' view, this improves the treatment and care for the residents. This subtheme involves including the participants' perceptions in the treatment process and constructive collaboration with GPs.

##### Including the participants' perceptions

All participants were committed to contributing to the residents' treatment. The participants wanted to share how they perceived residents' current state of health and emphasized that the AGIL team should consider these perceptions to facilitate a shared decision‐making process:

I find it meaningful to take on board the team which is around 24/7 and to show the resident: we are a part of the attending team.(Participant 10)

Most participants felt it was important that the AGIL team develop a holistic understanding of residents' conditions and observed that this was facilitated by integrating their perceptions in the treatment process. Several participants highlighted their bond of trust with old age psychiatrists from the AGIL team. The mutual trust facilitated expressing divergent positions in the treatment process and allowed to discuss and develop options for care together:

That I can communicate freely and openly with the doctor [of the AGIL team], that she has an open field of vision and is also open to something new and allows other points of view.(Participant 8)

One participant felt excluded from the treatment process and lacked information about one resident. She wished to receive professional input from the AGIL team to expand her scope of action when caring for this resident (see above, subtheme *expanding one's scope of action*). All other participants experienced partnership‐based collaboration with the AGIL team. Working together as partners may also contribute to the participants feeling valued in their professional role (see above, subtheme *valuing the experience and competence of nurses*).

##### Constructive collaboration with GPs

All participants described interprofessional collaboration with GPs. One third of the participants experienced that GPs accept the AGIL team and that they engage in constructive discussions. However, two thirds of the participants experienced friction in interprofessional collaboration with GPs. They reported that GPs had limited time and did not involve them in their clinical assessment or treatment decisions. Also, the participants felt that GPs did not actively include the AGIL team. Finally, some participants said that the old age psychiatrists of the AGIL team had no direct contact with the GPs. These participants found themselves in an intermediary role between GPs and the old age psychiatrists of the AGIL team. They were not comfortable in this position and would have preferred that GPs and the old age psychiatrists of the AGIL team interact directly with each other. Maintaining contact with each other could help with assigning tasks to different players. One participant experienced this clearly when the responsibilities of the AGIL team and the GPs were defined explicitly. This led to more structured and professional collaboration.

## 4 DISCUSSION

This study aimed to describe how nurses experience the involvement of AGIL in the care for older people experiencing mental health issues in nursing homes and to identify barriers and facilitators in the collaboration between nursing staff and the AGIL team. The present study shows for the first time that nurses in nursing homes felt relieved by having inclusive support. The results suggest that nursing staff appreciate services such as AGIL and feel accompanied and confident in their daily work. The interviews showed that nurses were able to expand their scope of action in challenging situations and to share responsibility together with the AGIL team.

There are few studies addressing the effects of outreach interprofessional mental health services in nursing homes. Fischer et al. (2011) showed that psychiatric consultation teams support the care of nursing home residents experiencing mental health issues. Koekkoek et al. (2016) showed that nurses experienced less work stress and that residents had significantly fewer psychiatric symptoms after consultation with a multidisciplinary psychiatric team (Koekkoek et al., 2016). Those studies did not, however, address nurses' experience. We found that the participants felt relieved from burden and believe that this reduces work stress, which likely translates into a lower intention to leave (Gaudenz et al., 2017; Kuo et al., 2014; Stewart et al., 2011). Some participants of our study expressed a lack of psychiatric knowledge and psychiatric nursing experience and expressed a need for further training. Muralidharan et al. (2019) showed that nurses in nursing homes are not adequately trained to care for residents diagnosed with severe mental disorders. Nurses who participated in this study appreciated that members of the AGIL team shared their knowledge, and they wanted to find new ways to cope with challenging situations. The literature shows that an increase in nurses' competencies is linked with higher job satisfaction and lower turnover intention (Aloisio et al., 2021; Tomietto et al., 2019). Thus, it may be beneficial to invest in further education and training for nurses.

Nurses in this study wanted to share responsibility for the care of the residents with the AGIL team and emphasized that a reliable contact person was important to them. This is consistent with the results of a study by Schärli et al. (2017), who suggest that high rotation and a lack of continuity hinder interprofessional collaboration. Furthermore, interprofessional collaboration is made easier if team members can work in proximity (Gurtner & Wettstein, 2019). Since AGIL is an outreach mental health service, there is a given physical distance between the nursing staff in nursing homes and the AGIL team. For nurses in nursing homes, a designated, accessible contact person might be more important. Some participants in this study reported having limited access to support during emergencies. This might indicate that the nursing homes are inadequately covered in case of psychiatric emergencies. Although responding to emergencies is not part of AGIL's remit, naming a contact person as well as informing nursing staff about absences and rotations of the AGIL team may make nurses feel better accompanied.

The present study showed that a partnership‐based collaboration is important to the nurses in nursing homes. Participants wanted to share their own perspectives and to be included in decision‐making processes. Several studies showed that it is pivotal to collaborate on equal levels, to value and to integrate different professions and their expertise (Collet et al., 2019; Schmitz et al., 2017; Weller et al., 2014). In line with these findings, nurses in this study expected that their experience and competence are valued by AGIL team members. Some nurses in the present study experienced friction in cooperating with GPs. Responsibilities were sometimes unclear. Fleischmann et al. (2016, 2017) investigated GPs' and nurses' views during nursing home visits. Their results show that nurses wanted to be involved in the treatment process, which is in line with the findings of our study. To improve the collaboration with GPs, Fleischmann et al. (2016) propose a structured course of action according to the requirements of nursing homes. Further studies showed that context‐based and defined workflows improve interprofessional collaboration (Gurtner & Wettstein, 2019; Schmitz et al., 2017). Considering the diversity of settings that the AGIL team and other outreach mental health services work in, it may be demanding to define these workflows on a case‐by‐case basis. Given that constructive interprofessional collaboration is linked with improved outcomes in patient care, this finding shows that there is potential for further development (Zwarenstein et al., 2009). Implementing defined workflows and responsibilities might improve interprofessional collaboration among the stakeholders.

Our results show that the AGIL team and the participants work in heterogeneous and unpredictable situations and settings, which are key characteristics of CAS (Pype et al., 2018). Based on CAS theory, a linear approach to practice is not reasonable. Rather, several attempts and joint development of individual strategies are needed (Pype et al., 2018). Our results relate to various aspects of CAS. The finding that nurses feel more confident when they are accompanied is consistent with the concept of ‘workplace learning’ (Pype et al., 2018, p.6). This concept is characterized as ‘emergent behaviour’ that may lead to team members acting more independently and may change interactions within a team. Both main categories imply that it is necessary to focus on interactions and relationships in healthcare teams (Khan et al., 2018). Empowering nurses and improving interprofessional collaboration can have beneficial effects on nurses and may also improve the care for the residents (Klug et al., 2019; van der Wolf et al., 2019).

## 5 IMPLICATIONS FOR NURSING PRACTICE

This study suggests that an interprofessional mental health service for nursing homes can be supportive to nurses caring for older people experiencing mental health issues in nursing homes. The following implications may help to further develop AGIL and other outreach interprofessional mental health services.

- Nurses in this study were committed to extending their knowledge about how to find new ways to cope with challenging situations. Outreach interprofessional mental health services should include training units and encourage exchanging knowledge in informal settings. In this way, AGIL and similar services may facilitate continuous learning, enable nurses to expand their scope of action, strengthen their clinical experience and empower nurses in their professional role.
- Nurses in this study wanted to share the responsibility for the care of residents with members of the AGIL team.The key elements to facilitate this are including the participants' perceptions in the treatment process, valuing and respecting their role as nurses, establishing a steady and reliable contact person and easing the workload of nursing staff.
- Outreach interprofessional mental health services such as AGIL should actively include nursing staff in the treatment of residents experiencing mental health issues. Context‐based processes and responsibilities of the involved stakeholders should be explicitly defined. This may improve interprofessional collaboration among outreach interprofessional mental health teams, nurses in nursing homes and GPs.

## 6 LIMITATIONS

The sample in this study included solely nursing staff. A larger sample involving all stakeholders, such as AGIL team members and GPs, would have helped to paint a broader picture. Also, we could not include team members of other outreach interprofessional mental health services because these are not established widely in Switzerland. This could limit the generalisability of our results. Ward service managers, who acted as gatekeepers, had high workloads that were more intense during the period of data collection due to the COVID‐19 pandemic (Riou et al., 2021). Due to high workloads, two participants seemed under pressure during the interviews and may have described their experiences in less detail than the other participants. Attaining an adequate sample size was demanding given those circumstances. Contrary to our initial intention to include only nurses with a tertiary education in the sample, we also interviewed two healthcare assistants. Healthcare assistants have an upper secondary education and bear a high level of responsibility in Swiss nursing homes.

## 7 CONCLUSION

Our results suggest that an outreach interprofessional mental health service such as AGIL contributes to relieving the burden on nursing staff caring for older people experiencing mental health issues in nursing homes. Our findings point out opportunities for further developing AGIL and similar outreach interprofessional mental health services.

## 8 RELEVANCE STATEMENT

To support nursing staff in the care for nursing home residents, the University Hospital of Psychiatry Zurich, Switzerland, established an outreach interprofessional mental health service. This study explored how nurses in nursing homes experienced the involvement of the service, and it identified barriers to and facilitators of interprofessional collaboration. Results show that nurses felt supported by the service and point out opportunities to further develop the service. The findings will help to improve interprofessional collaboration and to further develop this and other outreach interprofessional mental health services that aim to improve the care for residents experiencing mental health issues.

## CONFLICT OF INTEREST

Samuel Vögeli was part of the AGIL team when the study was conducted. He has established significant parts of AGIL and has been involved in its operations.

## ETHICS STATEMENTS

The Ethics Committee of the Canton of Zurich issued a declaration of non‐competence (req 2020–00854). The manuscript is based on interviews with nurses. No health‐related personal data were obtained. The risks for the informants were minimal.

## Data Availability

The data that support the findings of this study are available from the corresponding author upon reasonable request.
